# Extrapulmonary Clinical Manifestations in COVID-19 Patients

**DOI:** 10.4269/ajtmh.20-0986

**Published:** 2020-09-15

**Authors:** Aila Sarkesh, Amin Daei Sorkhabi, Elham Sheykhsaran, Farbod Alinezhad, Nader Mohammadzadeh, Nima Hemmat, Hossein Bannazadeh Baghi

**Affiliations:** 1 Immunology Research Center, Tabriz University of Medical Sciences, Tabriz, Iran;; 2 Infectious and Tropical Diseases Research Center, Tabriz University of Medical Sciences, Tabriz, Iran;; 3 Drug Applied Research Center, Tabriz University of Medical Sciences, Tabriz, Iran;; 4 Students’ Research Committee, Tabriz University of Medical Sciences, Tabriz, Iran;; 5 Department of Bacteriology, Faculty of Medicine, Tabriz University of Medical Sciences, Tabriz, Iran;; 6 Department of Virology, Faculty of Medicine, Tabriz University of Medical Sciences, Tabriz, Iran;; 7 Central Medical Laboratory of East Azerbaijan Province, Tabriz University of Medical Sciences, Tabriz, Iran

## Abstract

COVID-19 manifestations in symptomatic patients can be in the form of pneumonia, acute respiratory syndrome, and multiple organ dysfunction as well. Renal complications, gastrointestinal dysfunctions, endocrine system disorders, myocardial dysfunction and arrhythmia, neurological dysfunctions, dermatological symptoms, hematological manifestations, and thromboinflammation are among the reported extrapulmonary complications. Moreover, the presence of coagulopathy, excessive and dysregulated immune responses, and autoimmunity by COVID-19 patients is considerable. The pathogenesis of infection entails the entry of the virus via receptors on cells, principally angiotensin-converting enzyme 2 receptors. Direct virus damage coupled with indirect effects of viral infection including thromboinflammation, dysfunction of the immune system, and dysregulation of the renin–angiotensin system leads to multiple organ failure. This review outlines the extrapulmonary organ–specific complications and their pathophysiology and epidemiology.

## INTRODUCTION

In December 2019, the SARS-CoV-2 epidemic emerged in Wuhan and rapidly covered the whole world, becoming a pandemic. This novel viral infection may lead to severe viral pneumonia in some cases, causing respiratory failure, multi-organ and systemic dysfunctions due to sepsis, septic shock, and consequently death. The initial phase in the SARS-CoV-2 infection is the virus entry into the host cells. This process occurs, at least in part, through the interaction between the spike protein (S) of the virus and angiotensin-converting enzyme 2 (ACE2) receptors. Theoretically, any organ with ACE2 expressing cells is potentially susceptible to SARS-CoV-2 infection. Besides direct viral infection, indirect mechanisms such as thromboinflammation, dysfunction of the immune system, dysregulation of the renin–angiotensin system, and therapeutic effects lead to multiple organ dysfunction. These manifestations should be carefully considered in clinical settings in diagnosing and monitoring the manifestations to restrict a further person-to-person transmission and to extend therapeutic strategies for various system complications.

## SARS-CoV-2 PATHOGENESIS

### Direct mechanism of viral infection.

The initial step of infection is the virus entry. The spike subunit of SARS-CoV-2 binds to the receptor on the host cell. Spike protein includes two subunits, S1 and S2. S1 determines the host range and cellular tropism and facilitates virus attachment to the target cells. Also, the serine protease transmembrane protease, serine 2 (TMPRSS2) primes SARS-CoV-2-S for entry.^1^ Moreover, according to a study by Wang et al.,^1^ the SARS-CoV-2-S protein binds to cluster of differentiation (CD) 147 which is the transmembrane protein of the immunoglobulin family. Therefore, the immune system itself can be an entryway for SARS-CoV-2. These targets act on the initial phase of the SARS-CoV-2 infection. Hence, theoretically, any organ with ACE2 expressing cells is a target for SARS-CoV-2 infection.

### Indirect mechanisms of viral infection.

Besides direct invasion of SARS-CoV-2 to host cells, there are some indirect effects of viral infection that lead to multiple organ failure, including thromboinflammation, dysfunction of the immune response, and dysregulation of the renin–angiotensin–aldosterone system (RAAS).^2^

#### Dysfunction of the immune response.

The ensuing cytokine storm triggers a violent inflammatory immune response by high secretion of cytokines and chemokines, such as interleukin (IL)-1, IL-6, IL-8, IL-17, IL-17, chronic lymphocytic leukemia (CLL)-2, tumor necrosis factor-alpha (TNF-ɑ), granulocyte colony-stimulating factor (G-CSF), interferon gamma inducible protein (IP)-10, monocyte chemoattractant protein-1 (MCP1), and macrophage inflammatory protein (MIP).^3^ In COVID-19 patients’ sera with the need for intensive care unit (ICU) admission, there is a high concentration of G-CSF, C-X-C motif chemokine ligand 10, MCP1, MIP1A, and TNF-α. Furthermore, these cytokines are responsible for attracting neutrophils to the inflammatory sites.^4^ It seems that increased cytokine levels, especially IL-6, are associated with the worsened condition of patients.^3^ At first sight, innate immunity characterized by neutrophils and pro-inflammatory cytokines in the format of cytokine storm and is trying to limit the infection and overcome the virus. However, it leads to excessive inflammatory response with hazardous effects. Furthermore, SARS-CoV-2 infection can activate both nuclear factor kappa-light-chain-enhancer of activated B cells (NFkB) and signal transducer and activator of transcription 3 (STAT3), which in turn can activate the IL-6 amplifier, a mechanism for the hyperactivation of NFkB by STAT3, which leads to multiple inflammatory and autoimmune diseases.^5^

#### Thromboinflammation.

Angiotensin-converting enzyme 2 expression has been demonstrated in atrial and venous endothelium of different organs.^6^ Therefore, the invasion of SARS-CoV-2 to these cells results in infection-mediated endothelial injury which triggers thrombin production, inhibits fibrinolysis, and activates complement pathways. Hence, thromboinflammation and microvascular dysfunction occur.^6^

#### Dysregulation of RAAS.

Dysregulation of RAAS due to increased angiotensin II and decreased ACE2 can lead to a harmful inflammatory response. The abnormal functions of RAAS lead to another pathophysiological mechanism of SARS-CoV-2 infection–related, tissue damage. Angiotensin-converting enzyme 2 acts as a regulator of the RAAS pathway. Angiotensin-converting enzyme 2 also cleaves angiotensin I into inactive angiotensin 1–9 and cleaves angiotensin II into angiotensin 1–7, which has vasodilator, antiproliferative, and antifibrotic properties.^2^

## CARDIOVASCULAR SYSTEM MANIFESTATIONS

### Heart.

#### Prevalence and incidence.

There are positive correlations between the levels of cardiac biomarkers and the severity of COVID-19 infection, which indicate the prognostic value of these biomarkers.^7^ As the National Health Commission (NHC) of China announced, cardiovascular manifestations are even more tangible among patients rather than other clinical manifestations. It has been reported that 11.8% of COVID-19 patients without a history of cardiovascular disease present heart damage with elevated levels of troponin or cardiac arrest during hospitalization.^8^ A study on 75 hospitalized patients with SARS-CoV infection revealed that 40% of fatalities occurred following a myocardial infarction.^9^ Huang et al.^10^ demonstrated that SARS-CoV-2–associated myocardial injury occurred in five of 41 patients and was manifested as an increase in high-sensitivity cardiac troponin test (hs-cTnI) levels (> 28 pg/mL). Among these five patients, ICU management was required in four, indicating the severe nature of the myocardial injury presented by COVID-19 patients. According to a study on 187 COVID-19 patients by Guo et al.,^7^ it was demonstrated that the level of troponin was elevated in 27.8% of cases. During hospitalization, patients with elevated troponin T (TnT) levels developed more frequently complications such as acute respiratory distress syndrome (ARDS) (57.7% versus 11.9%), malignant ventricular arrhythmias (11.5% versus 5.2%), acute coagulopathy (65.8% versus 20.0%), and acute kidney injury (AKI) (36.8% versus 4.7%) than patients with normal TnT levels.^7^ Nonetheless, observations show that the mortality was significantly higher in patients with elevated plasma TnT levels than in patients with normal TnT levels (59.6% versus 8.9%).^7^ According to the NHC, among the people who died from COVID-19, 11.8% had substantial heart damage, with elevated troponin I levels or cardiac arrest during hospitalization.^8^ Laboratory results of COVID-19 patients indicated a high level of TnT and N-terminal pro-B-type natriuretic peptide (NT-proBNP). In these findings, the chest X-ray was normal, and the electrocardiogram (ECG) showed diffuse ST-segment elevation. Urgent coronary angiogram did not show coronary obstruction. Echocardiogram and cardiac magnetic resonance imaging showed evidence of myocardial inflammation, pericardial effusion, and poor left ventricular function, and nasopharyngeal swab with positive reverse transcription polymerase chain reaction (RT-PCR) results confirmed the diagnosis of COVID-19. These results highlight the importance of recognizing the cardiac manifestations of COVID-19 as an initial presentation, even with the absence of lower respiratory tract symptoms or radiological features of interstitial pneumonia.^11^

#### Pathophysiology.

According to the RNA-seq analysis of the human, more than 7.6% of myocardial cells have ACE2 expression.^12^ Human induced pluripotent stem cells (iPSC)-derived cardiomyocytes are susceptible to ACE2-mediated direct infection by SARS-CoV-2. Hence, the virus may induce detrimental cytopathic effects in these cells.^13^ These findings support the susceptibility of the heart tissue to be affected by the novel coronavirus. The most likely localization of the viral infection is in interstitial cells or macrophages infiltrating the myocardial tissue rather than localization in the myocytes.^14^ As heart function is associated with the RAAS, infection by SARS-CoV-2 gives rise to severe damages and dysfunction in the cardiovascular system.^9,15^ The inflammatory syndrome is mainly initiated by the induction of chemokines and cytokines such as interleukins, interferons (IFNs) and TNF-α. Cytokine storm depresses myocardial function through the activation of the neural sphingomyelinase pathway.^9^ Myocardial infarction in these patients is caused by inflammatory response syndrome, which principally might result in plaque rupture and thrombus formation.^16^ Infection-induced hypoxemia and vasoconstriction are other mechanisms that indirectly affect the heart, leading to ischemia in the heart muscles. COVID-19 can induce hypoxia by promoting ARDS or severe pneumonia. Furthermore, it has been demonstrated that hypoxia can result from the deteriorating oxygen transfer function of hemoglobin.^15^ A mismatch between oxygen supply and demand can lead to severe ischemia in heart tissue. It has been demonstrated that COVID-19 can contribute to cardiovascular manifestations with nonischemic mechanisms including fulminant myocarditis and cardiomyopathy.^9^ Hereupon, cardiomyopathy without any respiratory symptoms can be seen among COVID-19 patients.^9^ Studies have demonstrated that there is a correlation among the levels of TnT, C-reactive protein (CRP), and NT-proBNP.^17,18^ It indicates that myocardial injury in COVID-19 patients is associated with the severity of infection-caused inflammation and ventricular dysfunction. Regarding SARS-CoV infection and induced chronic cardiovascular manifestations, these kinds of damage lead to dysregulated lipid and glucose metabolism. It could be proven by elevated levels of free fatty acids, lysophosphatidylcholine, and lysophosphatidylethanolamine in serum analysis of patients with SARS-CoV infection.^8^ Because of the similarities in the pathogenicity of SARS-CoV-2 and SARS-CoV, these manifestations can also be expected in COVID-19 patients. Indeed, studies have shown that these patients have higher levels of the D-dimer, fibrin degradation products, and fibrinogen than a healthy population.^9^ These macromolecules increase the risk of venous thromboembolism (VTE) as well. Hence, abnormal levels of these macromolecules have been proven to be associated with the mortality in COVID-19 patients.^9^ Moreover, antiviral drugs may pose a hazard, such as cardiotoxicity, to the heart function. Hydroxychloroquine and chloroquine are known to induce arrhythmias.^19^

### Vascular system.

#### Prevalence and incidence.

Venous thromboembolism is a frequent cardiovascular or respiratory complication which occurs among hospitalized COVID-19 patients who are often elderly and immobile.^20^ These patients show signs of coagulopathy. Presently, the incidence of VTE is estimated at around 25% of hospitalized patients in the ICU for COVID-19.^21^ Data for both pulmonary embolism (PE) and deep vein thrombosis (DVT) are available from 16 studies, whereas six reported only on PE and seven only on DVT. Overall, the incidence of VTE was 26%, including PE with or without DVT in 12% and DVT alone in 14% of studies, 14 studies reported information on the extension of PE which involved the main trunk or lobar pulmonary arteries in 37.8% of cases, segmental arteries in 37.9%, and subsegmental arteries in 19.0% of cases.^21^ Thirteen studies reported on the extension of DVT which involved the proximal veins in 32.2% of cases and distal veins in 67.0% of cases.^21^ Similarly, findings show that Kawasaki-like disease and Kawasaki shock–like syndrome have been manifested by some COVID-19 patients.^22^

#### Pathophysiology.

Angiotensin-converting enzyme 2 is highly expressed by endothelial cells that line the vascular beds of different organs that can be directly infected by SARS-CoV-2.^6^ Recruitment of immune cells to a site of viral infection can induce endothelial dysfunction associated with apoptosis.^6^ Therefore, endotheliitis within different organs can be directly caused by a viral infection. Another vascular system manifestation that has been presented by pediatric patients is Kawasaki disease (KD).^23^ Kawasaki disease is a systemic vasculitis with unknown cause which occurs mostly in children younger than 5 years. In most KD cases, coronary artery aneurysms are observed.^24^ Additional vascular complication presented by COVID-19 patients is VTE which is common among hospitalized patients in ICU for COVID-19.^20^ Moreover, studies on the pathophysiology mechanism of VTE in COVID-19 patients suggest that the inflammatory condition of these patients contributes to the incidence of VTE.^20^ Previous studies on SARS and Middle East respiratory syndrome (MERS) indicate that excessive activation of the complement system consisting of elevated lactic dehydrogenase, the D-dimer, bilirubin coupled with decreased platelets (PLTs), mild anemia, and renal and cardiac injury is a possible mechanism which contributes to the development of VTE in COVID-19 patients.^20^

### Blood.

#### Prevalence and incidence.

Lymphopenia and increased levels of certain cytokines, such as IL-6, have been closely associated with the severity of the disease.^25^ Patients admitted to the ICU show a dramatic decrease in T cells, especially CD8^+^ T-cell count. In seven of the studies, patients with severe COVID-19 displayed a lower PLT count than those with milder forms.^25^ Thrombocytopenia is common in critically ill patients and usually suggests serious organ malfunction or physiologic decompensation.^26^ According to a study, the D–D levels were significantly higher in patients with severe COVID-19.^26^ In addition, the rise of the D–D level indicates secondary fibrinolysis conditions in COVID-19 patients and gives rise to the mortality rate.^25^ However, no significant difference in PLTs and activated partial thromboplastin time values between severe and mild patients was observed (–0.08, 95% CI = –0.34 to 0.18, = 60.5%; –0.03, 95% CI = –0.40 to 0.34, =79.5% respectively).^22^ Increasing values of the D–D and prothrombin time support the notion that disseminated intravascular coagulation (DIC) may be common in COVID-19 patients.^22^

#### Pathophysiology.

Lymphocytopenia is a hematological complication of COVID-19 patients which has multifactorial pathophysiology.^27^ There are some hypotheses for COVID-19–induced lymphocytopenia. Because ACE2 receptors play a vital role in the pathogenesis of SARS-CoV-2, the presence of ACE2 receptors on lymphocytes in oral mucosa, lungs, and digestive system facilitates direct viral invasion to these cells.^28^

Neutrophilia is another hematological complication presented by COVID-19 patients which is caused by hyperinflammatory and cytokine storm following infection.^28^

The most important hematological complication presented by COVID-19 patients, especially critically ill patients, is thrombocytopenia.^29^ There are some possible mechanisms for COVID-19–induced thrombocytopenia. SARS-CoV-2 infection can lead to thrombocytopenia by causing disturbances in PLT production. It can cause disturbances by overactivated immune responses and overproduction of inflammatory cytokines which has a destructive effect on progenitor cells of the bone marrow or by direct invasion to bone marrow stem cells.^30^ Moreover, dysregulated immune responses following SARS-CoV-2 infection may lead to an increase in autoantibodies which causes PLT destruction and thrombocytopenia. These autoantibodies are produced against glycoproteins expressed on the PLT surface which is followed by COVID-19–induced immune thrombocytopenic purpura.^24,30^ COVID-19–induced lung injuries play an important role in developing thrombocytopenia in these patients. Some studies indicate megakaryocytes and large cytoplasmic fragment participation in PLT production in pulmonary circulation which can be disturbed following COVID-19–induced lung injuries.^28^ Another mechanism for COVID-19–induced thrombocytopenia is the development of DIC in patients which results in overconsumption of PLTs.^28^ Disseminated intravascular coagulation is caused by the activation of the vascular endothelium, PLTs, and leukocytes, which results in dysregulated thrombin generation. The effects of the dysregulated generation of thrombin are further amplified by the inhibition of fibrinolysis and the impairment of natural anticoagulant mechanisms.^31^

## DIGESTIVE SYSTEM MANIFESTATIONS

### Gastrointestinal (GI) tract.

#### Prevalence and incidence.

**Figure 1.**
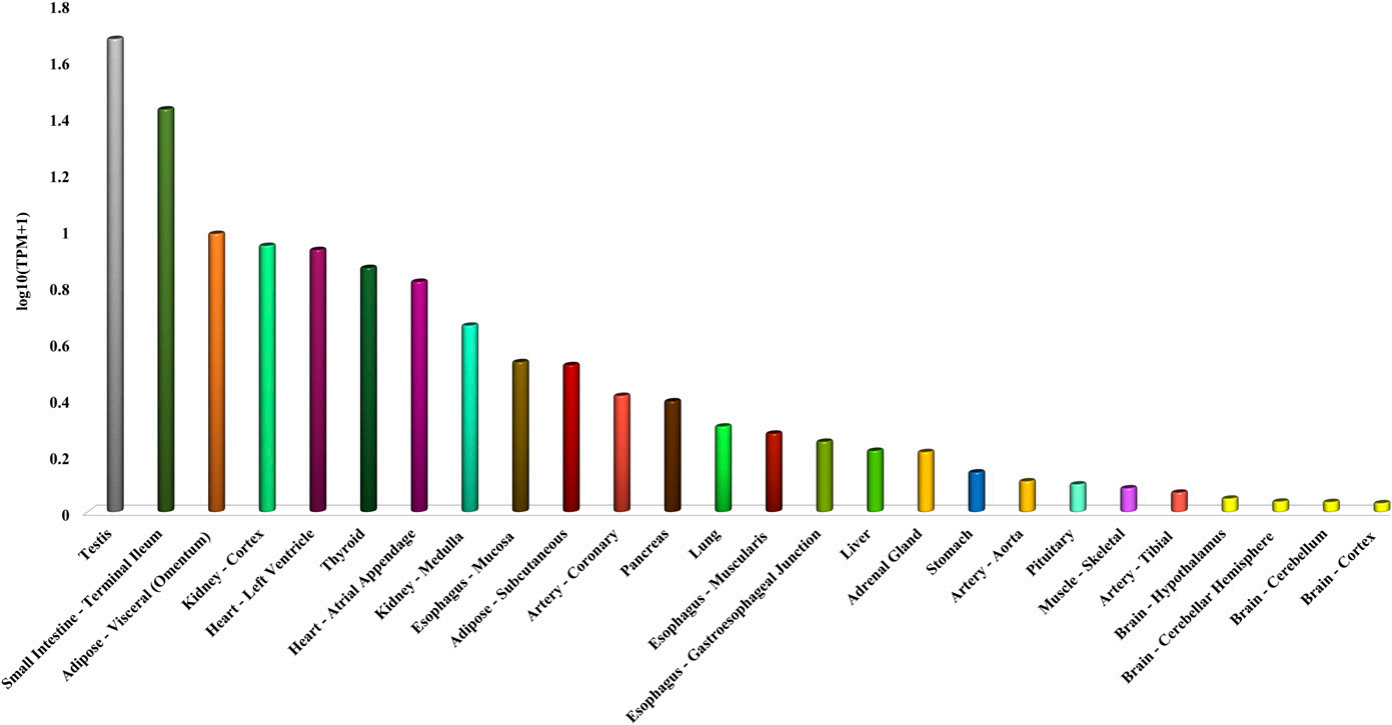
Angiotensin-converting enzyme 2 (ACE2) expressing cells of various organs. These data are provided from genotype-tissue expression (GTEx) project, as preprocessed values; these GTEx values are directly used in the below formula in lieu of the number of mapped reads and gene length. ACTB = beta-actin; TPM = transcripts per million.

Gastrointestinal symptoms may be the main evidence of COVID-19 in a certain subgroup of COVID-19 cases (Figure 1). According to the collected data from COVID-19 patients with or without GI symptoms, it has been revealed that GI symptoms during disease progression widely appear.^12^ The latest data from Wuhan showed that up to 79% of COVID-19 patients manifest GI symptoms such as diarrhea, loss of appetite, nausea, vomiting, abdominal pain, and GI bleeding during the onset of the disease and subsequent hospitalization. It is concluded that anorexia is the most frequent digestive symptom presented by adults (39.9–50.2%), whereas diarrhea is the most common symptom in adult patients and pediatric patients combined (2–49.5%), whereas vomiting is more common in children^32^; 3.6–15.9% of adult patients and 6.5–66.7% of pediatric patients manifest vomiting. Nausea is present in 1–29.4% of cases and GI bleeding in 4–13.7% of patients. Also, abdominal pain (2.2–6.0%) is more frequent in severe cases.^32^ Fang et al.^33^ reported a high GI symptom burden in both severely ill and stable patients, with an incidence of 85% and 79%, respectively. According to the Chinese population study by Tian et al.,^34^ anorexia was the most frequent digestive symptom in adults (30–50%), whereas the prevalence of diarrhea ranged from 2% to 50%. Some adults also presented vomiting, whereas GI bleeding and abdominal pain were found in more severely ill patients. The prevalence of GI symptoms in severely ill and non–severely ill patients is conflicting. A similar trend was observed in a large study on 1,099 patients by Guan et al.,^35^ where they reported no difference in the proportion of GI symptoms in severe versus non-severe cases of COVID-19.

$$\text{ACE}2\text{ expression}=\frac{\text{No}.\text{ of reads mapped to ACE}2\;/\text{length of ACE}2}{\text{No}.\text{ of reads mapped to ACTB}/\text{length of ACTB}}\times 1000.$$

According to the reports, correlation between the presence of the virus in the respiratory tracts and feces and the severity of the disease is not associated with the extended duration of viral RNA in stool samples.^36^ Therefore, in part with the respiratory transmission, a potency for fecal–oral transmission of SARS-CoV-2 has been reported as well.^37^ Findings state that, in some cases, SARS-CoV-2 has been cleared from the respiratory tract; however, the virus continues to replicate in the GI tract and can be shed through feces.^36^ Nevertheless, there are no certain data about the activation time of the virus in feces and no available conclusion about the SARS-CoV-2 continuous replication in the GI tract following the clearance of the virus from the respiratory tract.^36^

#### Pathophysiology.

The single-cell RNA-sequencing data obtained from the digestive system explain the presence of ACE2 expressing cells in the esophagus, ileum, stomach, and liver.^18,38^ Angiotensin-converting enzyme 2 staining is rarely seen in esophageal mucosa because the esophageal epithelium is mainly made of squamous epithelial cells, which express less ACE2 than glandular epithelial cells.^39^ Accordingly, it is demonstrated that ACE2 is highly expressed in the small intestine, particularly in proximal and distal enterocytes.^18,38^ Because enterocytes are directly exposed to the food and pathogens, they are highly susceptible to SARS-CoV-2 infection.^38^ It has been reported that ACE2 controls intestinal inflammation and diarrhea; therefore, the interaction between SARS-CoV-2 and ACE2 receptor might disrupt the ACE2 function, leading to diarrhea and other GI symptoms.^38^ However, the active viral replication and induction of type III interferons and inflammatory mediators in human enteroids might contribute to the development of GI symptoms in patients.^40^ According to the studies, ACE2-based strategies against the SARS-CoV-2 infection such as ACE2 fusion proteins should be accelerated into the clinical research and development for diagnosis, prophylaxis, or treatment. Moreover, some of the manifestations may occur because of side effects of treatments for diarrhea and may be related to the use of Arbidol, chloroquine phosphate, lopinavir, and remdesivir.^41^

### The liver and gallbladder.

#### Prevalence and incidence.

Regarding the research on 417 COVID-19 patients, 76.3% had abnormal liver tests and 21.5% had a liver injury during hospitalization.^42^ Studies have shown abnormal levels of alanine aminotransferase, aspartate aminotransferase (AST), and bilirubin in 14.8–53% of COVID-19 patients. Elevated levels of gamma-glutamyl transferase (GGT) and alkaline phosphatase have been observed in these patients.^43^ However, even in severe cases, significant liver injury is uncommon and liver dysfunction is mild, with only microvesicular steatosis in biopsy.^44^ It is known that elevated levels of aminotransferases are not specific to liver injuries. These elevations can result from the myositis induced by COVID-19.^44^ The elevation of AST was observed in eight (62%) of 13 patients in the ICU compared with seven (25%) of 28 patients who did not require care in the ICU.^10^ According to 12 studies, liver function abnormalities are observed in about 19% of patients.^45^

#### Pathophysiology.

COVID-19 can induce liver damage through direct and indirect interactions.^42^

##### Direct damage.

According to the single-cell–sequencing analysis, ACE2 receptor expression in liver tissue is only approximately 0.3%, and the specific expression of ACE2 in epithelial cells of the bile duct is 20 times higher than that in hepatocytes. Regarding the low expression of ACE2 by hepatocytes, direct liver damage requires further investigations.^46^ Serum GGT, as a diagnostic marker for cholangiocyte injury, has been found at elevated levels in up to 72% of severe COVID-19 patients.^47^ Hence, cholangiocytes are more likely to be targeted by SARS-CoV-2 and contribute to liver injury.^48^

##### Cytokine storm, hypoxia, and ischemia.

**Figure 2.**
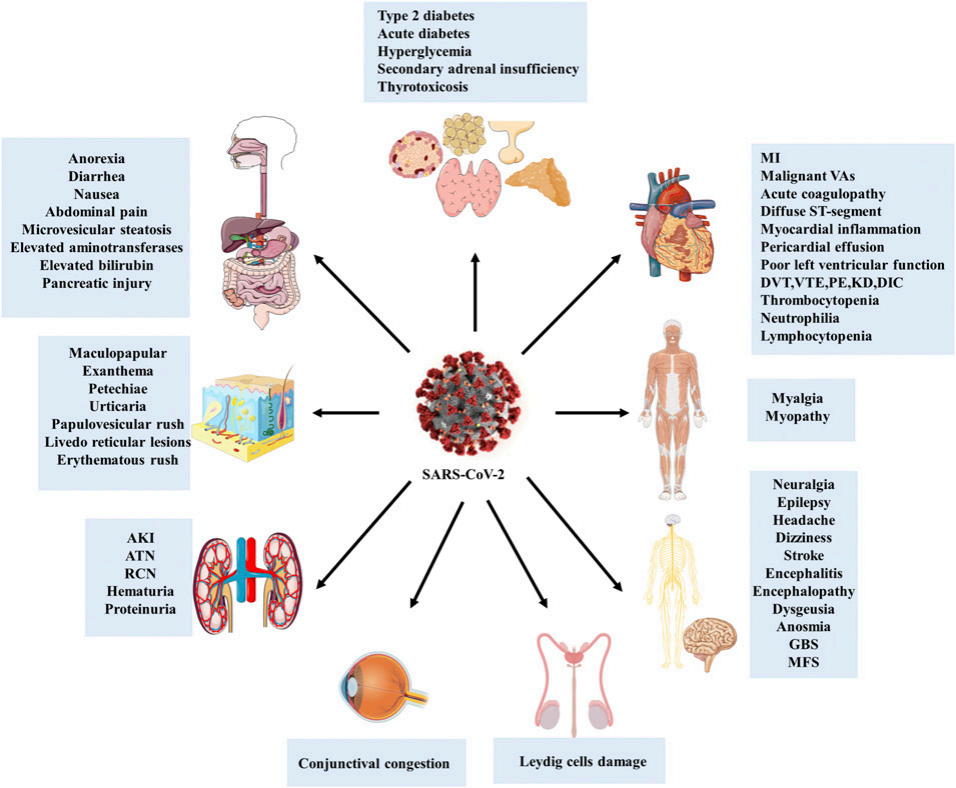
Extrapulmonary manifestations of SARS-CoV-2. Although COVID-19 is principally a respiratory disease, according to the studies, SARS-CoV-2 can infect different body sites through targeting angiotensin-converting enzyme 2 receptors, so the various infected organs manifest individual symptoms.

Following SARS‐CoV‐2 infection, a large number of immune cells may be overactivated and secrete excessive cytokines, and chemokines such as TNF‐α, IFN‐γ, IL‐6, and IL‐8. These immune responses are similar to those of other viral respiratory infections and are related to the intrahepatic cytotoxic T cells and Kupffer cells^44,46^ (Figure 2) which leads to ischemia, hypoxia, ARDS, and systemic inflammatory response syndrome (SIRS). Because of ischemia and hypoxia, lipid accumulation, glycogen consumption, and adenosine triphosphate depletion of hepatocytes can inhibit cell survival signal transduction which rapidly leads to hepatocyte death.^49^ In addition, pathological changes such as spleen atrophy and lymph node necrosis may be manifested. In severe cases of COVID-19, imbalanced immune responses have been observed, in which liver damages are accompanied by the coagulative and fibrinolytic pathways with the necrosis of liver cells and alterations in iron metabolism and macrophage activation.^44^

##### Recurrence of existing liver disease.

Different existing liver diseases influence liver injury in COVID‐19 patients. Nonetheless, interactions between preexisting liver disease and COVID-19 need to be further investigated. Patients with liver cirrhosis are more susceptible to infections because of their systemic immunocompromised status.^50^ For patients with chronic hepatitis B virus (HBV), who are in the immune tolerance phase, further investigations are needed to confirm whether these patients have active viral replication and persistent liver injury after coinfection with SARS‐CoV‐2 or not.^49^ Some underlying liver diseases, as well as chronic HBV infection, could be reactivated, and contribute to liver enzyme abnormalities in COVID-19.^51^

##### Drug hepatoxicity.

It is considered that hepatotoxicity can be induced following the use of some drugs. Lopinavir and ritonavir can cause abnormal levels of liver enzymes. Likewise, the combination of an overdose of lopinavir and ritonavir activates the endoplasmic reticulum stress pathway in the liver, inhibits the proliferation of hepatocytes, induces apoptosis of hepatocytes through the caspase cascade system, induces inflammatory reactions, and accelerates liver injury due to aggravating oxidative stress.^42,52^ Furthermore, biological drugs such as tocilizumab and baricitinib may lead to liver dysfunction.^53^

### The pancreas.

#### Prevalence and incidence.

Wang et al.^54^ showed that among 52 COVID-19 patients, 17% had a pancreatic injury. In this group, serum markers were mildly elevated (mean serum amylase 115 ± 25 U/L and serum lipase 71 ± 34 U/L). None of the patients had abdominal pain or clinically severe pancreatitis. Liu et al.^55^ also suggested 17% incidence of pancreatic injury among 67 severe COVID-19 cases. However, the injury was evident on computerized tomography (CT) scan in only 7.5% cases, mainly as focal pancreatic enlargement or pancreatic ductal dilatation. Incidence of pancreatic injury was low (1.9%) in patients with mild disease. Comparing the patients without pancreatic injury with those with pancreatic injury had a higher incidence of anorexia and diarrhea.^56^ They also had severe illness on admission, lower level of CD3^+^ T cells and CD4^+^ T cells, and higher levels of AST, GGT, creatinine, lactate dehydrogenase (LDH), and erythrocyte sedimentation rate than the patients without pancreatic injury.^54^

#### Pathophysiology.

Studies demonstrate the potential mild pancreatic injury patterns with pneumonia. These injuries might directly be related to the cytopathic effect of local SARS-CoV-2 replication. Moreover, these symptoms indirectly occur because of the harmful immune response induced by the virus or from secondary enzyme abnormalities.^54^ Acute pancreatitis can occur because of drug-induced injury either directly because of use of nonsteroidal anti-inflammatory drugs or glucocorticoids and indirectly through tocilizumab-induced hypertriglyceridemia.^55,57^

## NERVOUS SYSTEM MANIFESTATIONS

### The central nervous system (CNS).

#### Prevalence and incidence.

Several studies have highlighted CNS manifestations including headache, which is the most common CNS complications with the prevalence varying from 6.5% to 23%, and the mean prevalence of 8% among COVID-19 patients.^58^ Wang et al.^59^ showed that among 138 hospitalized COVID-19 patients, 13 had dizziness and nine had a headache. Similarly, those patients in the ICU were more likely to report dizziness. Furthermore, a case series research revealed that three critically ill patients with COVID-19 presented multiple cerebral infarctions, confirmed by brain CT scan, 10–33 days after the onset of the initial symptoms of the disease.^60^ A retrospective observational study from Wuhan, China, found 11 (5.0%) of 221 patients developed acute ischemic stroke.^61^ Older individuals, especially those with preexisting chronic medical conditions, are at high risk of impaired consciousness or delirium in the setting of acute infections. These patients, who are prone to experience COVID-19 severely, may present the encephalopathy and confusion. Confusion was reported in 9.0% of COVID-19 patients mostly in those with poor prognosis.^62^ Viral infections such as COVID-19 may provoke the occurrence of cerebrovascular diseases such as acute ischemic stroke.^5,16^ It may be caused by the downregulation of natural anticoagulant mechanisms by inflammatory mediators and disturbance of the coagulation system. In this regard, similar to MERS, disruption of the coagulation system is reported in COVID-19 patients.^63^

#### Pathophysiology.

Viral infections can lead to serious structural and functional damage to the nervous system.^64^ It has been proposed that SARS-CoV-2 enters through the olfactory nerve and reaches the brain.^65^ Through this pathway, the virus enters into the peripheral nerves and thereafter spreads into the specific brain areas including thalamus and brain stem.^66^ Indeed, the most direct pathway from the periphery to the brain is the olfactory neuroepithelium within the nasal cavity, where the cells of olfactory sensory neurons send their axons into the CNS to synapse with dendrites of mitral neurons within the olfactory bulb.^64^ Although the actual mechanism of virologic control is still unknown, studies indicate that acute immune response is specialized at this site, which promotes interaction between immune cells and neural cells, and recruits leukocytes which influence viral clearance.^64^

**Figure 3.**
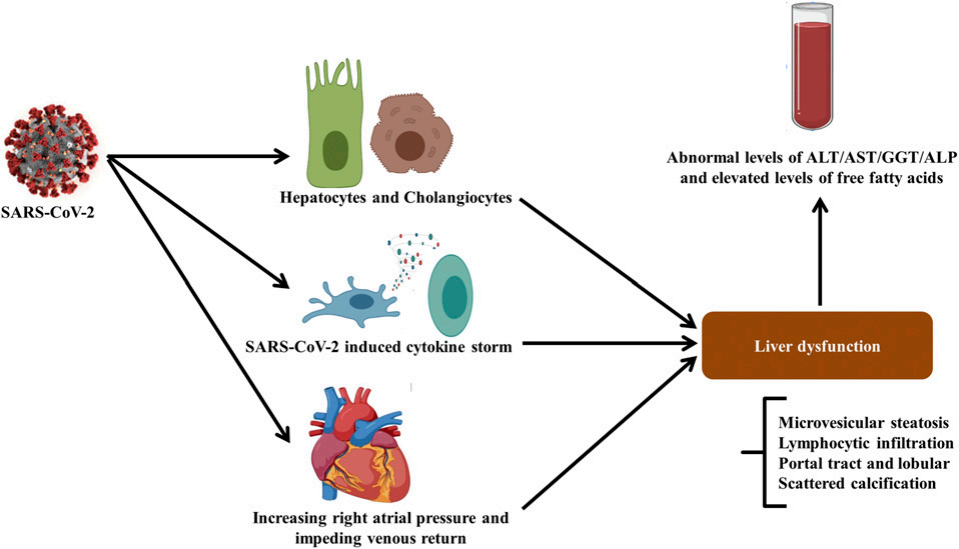
Mechanism of liver injury caused by COVID-19. SARS-CoV-2 infection may lead to liver injury through different mechanisms including direct viral toxicity, SARS-CoV-2–induced cytokine storm SIRS and increased right atrial pressure and impeding venous return effect which result in liver damage. These liver injuries can be detected by abnormal levels of alanine aminotransferase, aspartate aminotransferase, gamma-glutamyl transferase, alkaline phosphatase, and free fatty acids according to serum analysis.

Autopsy results of hospitalized COVID-19 patients with neurologic manifestations demonstrated the hyperemic and edematous properties for the brain tissue and degenerated condition for some neurons.^64^ Because of the virus entry to neuroplasm via the ACE2 receptors, SARS-CoV-2 can induce neuronal death through apoptosis, and in rare cases, autophagy might happen.^67^ It is well studied that ACE2 is expressed by brain cells (Figure 3) particularly in the brain stem and in regions that are responsible for the regulation of cardiovascular function. These regions include subfornical organs, paraventricular nucleus, the nucleus of the tractus solitarius, and rostral ventrolateral medulla.^64^ However, the existence of ACE2 receptors on host cells is not sufficient to make host cells susceptible to the virus entry, and other pathways for viral entrance must be considered. Hypoxia injury refers to the diffuse alveolar interstitial inflammatory exudation, edema, and the formation of transparent membranes followed by virus replication.^64^ It leads to the alveolar gas exchange disorders which cause hypoxia in the CNS. The hypoxia in COVID-19 patients can result in damages to the nervous system.^64^ The pathophysiology of severe viral infection is linked to the development of a SIRS. Systemic inflammatory response syndrome can be initiated in severe pneumonia followed by coronavirus infection. SARS and COVID-19 result in fatalities mostly due to the multiple organ failure caused by virus-induced SIRS or SIRS-like immune disorders. Furthermore, the activation of immune cells in the brain causes chronic inflammation and brain damage.^64^ The inflammation may induce symptoms related to the muscles, including fatigue, limb aches, and mild elevation of serum kinase levels.^68^ Angiotensin-converting enzyme is a cardio-cerebral vascular protection factor existing in a variety of organs, including the nervous system and skeletal muscles. It plays a major role in the regulation of blood pressure and anti-atherosclerosis mechanisms. Because ACE2 is the main target of SARS-CoV-2, binding to ACE2 receptors may give rise to abnormally elevated blood pressure and an increased risk of a cerebral hemorrhage. Chemoreceptors and mechanoreceptors in the lungs and respiratory tract send sensory information to the solitary nucleus, whereas nerve supply to blood vessels, airway smooth muscle, and glands are efferent fibers derived from the solitary nucleus and the nucleus ambiguous.^67^ These connections indicate that the respiratory syndrome experienced during SARS-CoV-2 infection may be caused by malfunction of the brain stem.^67^ Furthermore, SARS-CoV-2 infection destroys the blood–brain barrier, and secondary intracranial infections may cause headaches, projectile vomiting, visual loss, and limb convolution in patients with severe COVID-19 symptoms.^64^ Homeostasis characteristics of the cells in the CNS contribute to the continued existence of the virus.^64^

##### Nonspecific neurological manifestations.

Few nonspecific neurological symptoms have been reported in COVID-19 patients with varying frequency. These include dizziness 16.8%), headache (13.1%), muscle injury leading to myalgia and increased serum creatine kinase (CK) (10.7%), neuralgia (2.3%), epilepsy (0.5%), and ataxia (0.5%)^69^(Figure 3).

##### Stroke.

There are multiple evidence in the literature indicating that COVID-19 increases the risk of both venous and arterial thrombosis. COVID-19 patients with a new onset of ischemic stroke are more likely to have a severe SARS-CoV-2 presentation. COVID-19 patients manifest elevated right atrial pressure and impeding venous return which could contribute to the hepatic congestion.^44^ Mechanisms of SARS-CoV-2–induced liver injuries are not completely well understood; however, some of the features of coronaviruses studied on previous studies on MERS-CoV and SARS-CoV are considerable.^44^

##### Encephalitis and encephalopathy.

COVID-19 patients may present deterioration in conscious level and headache. This could be either followed by CNS infection or toxic encephalopathy.^70^ Encephalitis refers to the inflammatory lesions in the brain parenchyma which is accompanied by the neuronal damage and nerve tissue lesions.^71^ Moreover, infectious toxic encephalopathy is a type of reversible brain dysfunction syndrome that may occur because of SARS-CoV-2 infection, yet further investigations are required.^71,72^ Surprisingly, a rare form of encephalopathy called acute hemorrhagic necrotizing encephalopathy has been reported.^73^

### Peripheral nervous system.

#### Prevalence and incidence.

Clinical studies on COVID-19 patients have shown the occurrence of sudden anosmia and fatigue without other symptoms like fever and cough in the early stages^74^ Lechien et al.^75^ analyzed a total of 417 mild-to-moderate COVID-19 patients for the olfactory and gustatory dysfunctions, in which 357 patients (85.6%) had olfactory dysfunction, and among those, 284 patients (79.6%) and 73 patients (20.4%) showed anosmia and hyposmia, respectively. Furthermore, 12.6% of these patients were phantosmic, and 32.4% of patients were parosmic. Some rare cases of COVID-19 patients also present autoimmune disorders including Guillain–Barre syndrome and Miller Fisher syndrome.^64^

#### Pathophysiology.

##### Guillain–Barre syndrome.

Guillain–Barre syndrome (GBS) is a progressive, ascending, symmetrical flaccid limb paralysis, along with areflexia or hyporeflexia with or without cranial nerve involvement which can progress over days to weeks. The onset of GBS occurs approximately a week after the onset of SARS-CoV-2 infection. The postinfectious mechanism of GBS is supported by the finding of autoantibodies that result from an immune response directed to an epitope of the infectious agent that then cross-reacts with a structurally similar component of peripheral nerve. It leads to delayed immune-mediated damage to a peripheral nerve. It is yet unclear whether COVID-19 induces the production of antibodies against these specific gangliosides.^76^

The attachment of SARS-CoV-2 to cell surfaces is mediated by the viral spike (S) protein, which besides ACE2 binds to gangliosides containing sialic acid residues, including the N-acetylgalactosamine (GalNAc) residue of monosialotetrahexosylganglioside (GM1).^77^ It has been suggested that cross-reactivity between the viral protein–associated gangliosides and peripheral nerve gangliosides may result in molecular mimicry. The mechanism of nerve damage may be primarily facilitated by T‐cell activation and release of inflammatory mediators by macrophages.^77^ However, it is also possible that GBS cases have been incorrectly attributed to critical illness neuromyopathy.^78^

##### Miller Fisher syndrome.

Miller Fisher syndrome is an acute peripheral neuropathy that develops after exposure to viral, bacterial, and fungal pathogens.^79^ It is a rare disease and is observed in 5% of all GBS cases.^24^ Miller Fisher syndrome is often immune-mediated and associated with anti-ganglioside Q1b (GQ1b) antibodies, manifested by a triad of ophthalmoplegia, gait ataxia, and areflexia. Ophthalmoplegia is due to the involvement of cranial nerves III, IV, or VI. Ataxia might occur because of cerebellar involvement, and areflexia is due to the lower motor neuron involvement. Miller Fisher syndrome has been described as a very rare manifestation of COVID-19.^80^

##### Olfactory and taste dysfunction.

According to the previous research on coronaviruses, it is not surprising to diagnose anosmia in COVID-19 patients. Regarding extensive contributions of smell and taste senses, these patients also confront the difficulty of detecting flavors.^81^ According to the studies, the occurrence of inflammation in the olfactory nerves is more common than structural damage.^74^ This inflammation following viral infection can be caused by some viruses including rhinovirus, parainfluenza, Epstein–Barr virus, and some coronaviruses.^81^ Viruses can infect peripheral neurons and enter into the CNS through the olfactory bulb. It has been proved that the novel coronavirus antigens could be detected within the olfactory bulb.^81^ In animal models, it has been shown that coronaviruses that reach the CNS through olfactory bulb can induce demyelination and stimulate T-cell–mediated autoimmune reactions.^82^ It can lead to neurological damage to the CNS which might be followed by anosmia and dysgeusia in COVID-19 patients; anosmia can also be caused by swelling in the nose and sinuses due to chronic sinusitis.^82^ Some investigations claim that the proportion of ACE2-expressing cells in nasal and oral tissue is comparable to the lung tissue.^83^ It has been illustrated that olfactory epithelial support cells, stem cells, and nasal respiratory epithelium express TMPRSS2 and ACE2 genes by which SARS-CoV-2 can enter into them.^83^ These findings can support the idea that the nasal and oral tissues are the first tissues susceptible to be infected by SARS-CoV-2. Any dysfunction in the olfactory system will only be temporary because of its structural design and regenerative stem cells. These stem cells can generate new olfactory nerve cells to replace the damaged ones, and depending on the severity of inflammation, anosmia can be solved within a few days to weeks.^74^ According to the findings of a study on 88 patients with COVID-19 in the condition that 59 patients were able to be interviewed, 20 patients presented at least one of olfactory or taste disorders, and 12 patients presented these symptoms before admission to the hospital, and among them, taste alterations were more frequent.^74^ Another study demonstrated that among 357 patients, approximately 304 (85.6%) had olfactory dysfunction, 284 (79.6%) were anosmic, and 73 (20.4%) were hyposmic.^81^ It is considered that IFNs are also involved in the onset of taste disorders.^84^ IFNs are highly induced during a viral infection, and their receptors are highly expressed in taste epithelium compared with the surrounding non-gustatory epithelium.^84^ Inflammatory cytokines such as IFNs can cause apoptotic cell death as well. This can lead to the destruction of the taste bud cells, and they can be followed by the development of taste dysfunction.^85^ Studies on animal models demonstrated that ACE2 expression by the nucleus of the solitary tract and its role as a neuroinvasive route by continuous local or retrograde vagal axonal transport can be a central cause of dysgeusia.^85^

## OCULAR SYSTEM

### Prevalence and incidence.

According to a meta-analysis by Loffredo et al.,^86^ conjunctivitis in confirmed COVID-19 patients was 1.1% on average; in severe cases, it was 3% and 0.7% in non‐severe COVID-19 patients. Some studies on COVID-19 cases demonstrated conjunctivitis as an initial symptom of COVID-19,^87^ and recent research on 534 COVID-19 patients demonstrated that three patients presented conjunctival congestion as an initial symptom.^88^ Redness and excessive tearing were observed as well.^88^ Regarding a study on 38 COVID-19 patients, conjunctival swabs of two patients had been detected to be positive in the real-time quantitative reverse transcription (qRT-PCR) assay for SARS-CoV-2. However, 12 of them presented ocular manifestations.^89^

### Pathophysiology.

Studies on ocular tissue have shown that ACE2 is highly expressed by the human cornea and conjunctival tissues.^87^ This is something of a pitfall that mucosal surface of epithelia of the human eye is directly exposed to the outside environment. Hence, it can be infected by respiratory pathogens. The nasolacrimal system is an anatomical bridge between ocular and respiratory tissues.^90^ It facilitates the transfer of the virus from ocular tissue to the respiratory tract and vice versa; therefore, SARS-CoV-2 might spread to the lungs and cause respiratory system failures using the nasolacrimal route. Eyes have the potency to be directly infected by several viruses and at the same time can provide a route for the extraocular spread of the virus.^90^ Ocular clinical symptoms in COVID-19 patients generally include dry eyes, foreign body sensation, and blurred vision. However, it is considered that these symptoms could be related to the patients’ short distance reading and overuse of electronic devices during their hospitalization.^88^ According to the clinical observations, there is a high possibility for ocular transmission of SARS-CoV-2.^87^ Previous studies on SARS-CoV have shown that this virus is presented in tear samples as well.^91^ It supports the idea of the transmission of viruses through ocular tissue and secretions. Hereupon, hand–eye contact is considered as a risk factor for being infected by the novel coronavirus. Hence, wearing goggles and preventing hand–eye contact should be taken seriously by the public and in particular healthcare workers, to prevent further outbreaks of COVID-19.^91^ Also, these studies provide new strategies for the diagnosis of COVID-19 in early stages by analyzing conjunctival swabs and tear samples.^91^

## URINARY SYSTEM MANIFESTATIONS

### Prevalence and incidence.

Chronic kidney disease and AKI are seen in COVID-19 hospitalized patients. Further reported complications included acidosis and alkalosis.^92^ According to studies on kidney function of 59 patients with COVID-19, 28 of them were diagnosed as severe cases and three of them died. It was reported that 63% of patients present proteinuria. Besides, elevated levels of serum creatinine and urea nitrogen were found in 19–27% of the patients. On the other hand, CT of 27 cases showed inflammation and edema of renal parenchyma.^93^ Autopsy results of 26 COVID-19 patients showed diffuse damage in proximal tubules with the loss of brush border, vacuolar degeneration, and even necrosis.^94^

### Pathophysiology.

Detection of coronavirus in the kidneys and urine of COVID-19 patients supports the theory that the virus can directly damage the kidneys. Viral infection can induce tubular damage through the deposition of the *Mycobacterium avium* complex (MAC) complex on tubules and infiltration of CD68^+^ macrophages in the tubule interstitium.^95^ Besides direct infection, indirect mechanisms potentially lead to tubular injury, cytokine storm syndrome, shock/hemodynamic instability, rhabdomyolysis, and hypoxia of kidney tissue.^96^ Acute kidney injury may occur as a result of intrarenal inflammation, increased vascular permeability, volume depletion, and cardiomyopathy leading to cardiorenal syndrome type 1^97^(Figure 1). Cytokine release syndrome includes systemic endothelial injury and clinically manifests as pleural effusions, edema, intra-abdominal hypertension, third space fluid loss, intravascular fluid depletion, and hypotension.^97^ Extracorporeal oxygenation, invasive mechanical ventilation, and continuous kidney replacement therapy can also contribute to cytokine production.^97^ There is a close relationship between alveolar and tubular damage. According to the recent findings in ARDS, it is concluded that there is a probability of bidirectional alveolar–tubular damage in COVID-19 patients.^98^ Injured renal tubular epithelium promotes the upregulation of IL-6, which is associated with higher alveolar-capillary permeability and pulmonary hemorrhage.^99^ The fluid expansion increases alveolar-capillary leakage and AKI. It worsens renal vein congestion leading to renal compartment syndrome.^100^ The possible mechanism of renal disturbances presented by COVID-19 patients is dehydration which has various consequences on the kidney. It causes a reduction in the glomerular filtration rate and AKI. If the volume depletion is not severe, it can be reversed by hydration, but if ischemia persists like in a shock, acute tubular necrosis may occur.^101^ Furthermore, rhabdomyolysis and hypoxia are other possibilities.^101^ I, direct invasion of SARS-CoV-2 to renal tubular cells, interstitium, or glomeruli is possible, which confirms previous evidence of the direct cytopathic effect of the virus on renal cells.^101^

## REPRODUCTIVE SYSTEM MANIFESTATIONS

### Prevalence and incidence.

According to a recent study, 81 COVID-19 male patients’ total testosterone (T) was lower, whereas serum luteinizing hormone (LH) was significantly higher than that of 100 age-matched healthy men. The serum T:LH ratio was also significantly lower in COVID-19 patients and was negatively associated with disease severity.^102^

### Pathophysiology.

The existence of ACE2 receptors on the testicular cells including spermatogonia, Leydig, and Sertoli makes these cells a target for SARS-CoV-2 infection.^102^ To further characterize ACE2-positive cells in human testis, gene ontology (GO) enrichment analysis was performed to determine which biological processes are enriched within either spermatogonia or Leydig and Sertoli cells by comparing ACE2-positive cells with ACE2-negative cells. Twenty-four GO terms associated with viral reproduction and transmission were evaluated, which were positively enriched in ACE2-positive spermatogonia and include viral gene expression.^18^

A study on 12 COVID-19 patients demonstrated the absence of viral RNA in testicular biopsy tissues.^103^ It indicates that SARS-CoV-2 cannot directly infect testes or the male genital tract even in the acute phase; therefore, no evidence shows that the novel coronavirus can be sexually transmitted from males.^103^ Other studies indicated that serum LH in males with COVID-19 infection could be significantly increased; however, the ratio of T to LH and the ratio of follicle-stimulating hormone (FSH) to LH are dramatically decreased.^102^ Furthermore, regarding the serum analysis, elevated levels of prolactin (PRL) have been reported. Clearly, elevated levels of PRL lead to pituitary suppression.^102^ Hence, decreased levels of gonadotropins are expected. In COVID-19 patients, the level of LH in serum is reported to be increased as well. The elevated level of LH and decreased level of T (leading to low T/LH ratios) are more likely to be caused by testes dysfunctions such as the possible damage to Leydig cells.^102^ On the other hand, the levels of FSH, estrogen, and the ratio of testosterone: estrogen are not different between COVID-19 patients versus control groups. Follicle-stimulating hormone secretion is mainly suppressed by inhibin B which is secreted by Sertoli cells, and estradiol normally comes from the peripheral aromatization of androgens. Therefore, it seems that Sertoli cells are less disturbed than Leydig cells.^102^ The correlation between serum T:LH and the main clinical characteristics of patients with COVID-19 have been analyzed as well. The results indicated that CRP which is an acute phase protein and produced by the liver is significantly associated with the T:LH ratio. Elevated CRP level is accompanied by the abnormal production of cytokines such as interferon which leads to dysfunction of testes and spermatogonia.^102^

## DERMATOLOGICAL MANIFESTATIONS

### Prevalence and incidence.

According to research on 88 COVID-19 patients in Italy, 20.4% of patients developed cutaneous manifestations.^104^ These manifestations mostly consist of maculopapular exanthema, papulovesicular rash, and acral red–purple papules. However, rarely urticarial, livedo reticularis lesions and petechiae have been observed.^105^ Superficial perivascular dermatitis and dyskeratotic keratinocytes have been most commonly described from a histopathological analysis of skin rashes.^2^ These manifestations are generally localized on the trunk, but in some cases, they have been observed on the hands and feet.^105^ According to a case study, 69.4% of COVID-19 patients present these manifestations after the onset of respiratory symptoms and 12.5% of them at the onset of COVID-19 symptoms.^105^

### Pathophysiology.

According to studies, in most cases, the severity of cutaneous lesions was not correlated with COVID-19 severity.^105^ It is considered that viral rashes and drug reactions are clinically and histologically similar, and it is difficult to determine whether these manifestations are related to viral infection or not.^106^ Also, it has not been well confirmed that if these dermatological symptoms are caused by a skin infection or the consequences of respiratory infection.^106^ According to some theories, it is postulated that viral particles in the cutaneous blood vessels in COVID-19 patients can lead to lymphocytic vasculitis which can be followed by the production of cytokines and damage to the blood vessels in the skin.^105^ Following a viral infection, Langerhans cells migrate to the regional lymph nodes to induce dendritic cells. It can result in vasodilation and spongiosis, giving rise to the cutaneous manifestations.^105^ SARS-CoV-2 can induce hypoxia leading to the accumulation of deoxygenated blood in venous plexus. This accumulation can cause livedo reticularis-resembling manifestations.^105^ Cutaneous adverse events of antimalarials include cutaneous eruptions such as acute generalized exanthematous pustulosis, urticaria, pruritus, dry skin, rashes, flares of psoriasis and exfoliating lesions, Stevens‐Johnson–like syndrome, mucocutaneous dyspigmentation, alopecia, and bleaching of hair.^107^ Azithromycin can induce cutaneous severe skin reaction associated with fever, angioedema, skin pain, and generalized red or purple skin rashes. Regarding the asymptomatic features of COVID-19, these manifestations can act as an indicator for early diagnosis and prevent further outbreaks of this disease. According to a case report of a COVID-19 patient with cutaneous manifestations, serology tests against anti–SARS-CoV-2 antibodies were negative. However, based on PCR testing of the skin, SARS-CoV-2 at low copy numbers was detected.^108^ Hence, SARS-CoV-2 PCR testing of skin biopsy samples can be used as an additional diagnostic tool.

## MUSCULOSKELETAL SYSTEM MANIFESTATIONS

### Prevalence and incidence.

Given the results of clinical trials, COVID-19 patients manifest limb pain and several muscular disorders. These disorders include critical illness myopathy, acute quadriplegic myopathy, thick filament myopathy, and necrotizing myopathy.^109^ Myalgias have been reported in up to half of the patients with SARS-CoV-2 infection. Serum CK level elevations depend on the severity of the disease, ranging from mild to frank rhabdomyolysis. Furthermore, myositis and myasthenia gravis are both autoimmune diseases that might coexist.^110^

### Pathophysiology.

COVID-19–induced musculoskeletal manifestations can develop through direct and indirect mechanisms. In direct mechanisms, SARS-CoV-2 directly targets ACE2 expressed cells in the musculoskeletal system.^109^ Studies on ACE2 expression of the musculoskeletal system indicated skeletal muscle, synovium, and cortical bone as a potential site of direct SARS-CoV-2 infection.^109^ The indirect mechanism of COVID-19–induced musculoskeletal manifestations is caused by overactivated immune responses and COVID-19–induced cytokine storm. Elevated levels of pro-inflammatory molecules including IFN-g, IL-1b, IL-6, IL-17, and TNF-a can directly impact skeletal muscle by muscle fiber proteolyzing and disturbing protein synthesis.^109^ Furthermore, corticosteroids that are used for the reduction of inflammation in COVID-19 patients can induce musculoskeletal impairments.^109^ Reduced bone mineral density has been also reported in patients as an adverse effect of corticosteroid which is dependent on the extent and duration of treatment with corticosteroids.^109^

Critical illness myopathy is typically a non-necrotizing diffuse myopathy associated with fatty degeneration of muscle fibers, fiber atrophy, and fibrosis, and may represent an antecedent to acute necrotizing myopathy. This is distinguishable by the extensive myonecrosis with the vacuolization and phagocytosis of muscle fibers and is related to multiple organ dysfunction.^67^ Nevertheless, these functional disabilities in the muscles can be a result of ICU-acquired muscle loss and weakness and not due to the infection. The limb pains are associated with the complications in their large blood vessels which lead to a mismatch between blood supply and demand. Therefore, any mechanism that causes ischemia can be followed by limb pain. Ischemia can be caused by cardiopulmonary or vascular manifestations of the novel coronavirus. In some clinical trials performed on COVID-19 patients, lower limb pains are accompanied by the absence of dorsalis pedis, posterior tibial pulses, and initial skin marbling of the forefoot. These demonstrations can be caused by the thrombotic obstruction of the tibial arteries of the lower limb.^67^ Furthermore, myositis is another disorder in COVID-19 patients. There is a report about myositis in a patient who was not under medications and suddenly developed diffuse myalgias and proximal lower limb muscle weakness, causing him to fall. After a while, the patient was afebrile and did not present any upper or lower airway symptoms; however, after some days, the test for SARS-CoV-2 was positive. In this patient, the subsequent association of myositis followed by the interstitial pneumonitis led to the hypothesis of autoimmune myositis. However, all the immunological tests looking for any forms of myositis were negative.^111^ In conclusion, although COVID-19 manifestations are frequently presented in upper and lower airways, regarding this report, it can be revealed by the acute myositis. Because the association of muscle inflammation with interstitial pneumonia can be seen in either COVID-19 or autoimmune myositis, this differential diagnosis should be considered by clinicians.^111^

## ENDOCRINE SYSTEM

### Prevalence and incidence.

A study has been showed that 52 (61.9%) of 84 COVID hospitalized patients presented thyroid function abnormalities.^112^ Another study with 658 hospitalized COVID‐19 patients confirmed that 42 (6.4%) of 658 patients manifested ketosis on admission with no obvious fever or diarrhea.^113^ This report suggests that SARS-CoV-2 infection can cause ketosis per se in nondiabetic persons and may increase the risk of ketoacidosis in patients with diabetes.^113^ Regarding a study, up to 17% of COVID-19 hospitalized patients had evidence of some pancreatic injury (elevated amylase and lipase) and hyperglycemia postulated to be due to β-cell injury or as a result of severe systemic illness.^114^

### Pathophysiology.

#### Endocrine pancreas manifestations.

As ACE2 is highly expressed in both the exocrine glands and islets of the pancreas, therefore, endocrine pancreatic injury is expected.^55^ Angiotensin-converting enzyme 2 converts angiotensin II into angiotensin 1–7. When the virus blocks ACE2, angiotensin II degradation is suppressed and its level increases. Conversely, angiotensin 1–7 level decreases. Angiotensin II increases insulin resistance and beta-cell damage; whereas angiotensin 1–7 prevents insulin resistance. Furthermore, according to the serum analysis, COVID-19 patients with pancreas dysfunction present abnormalities in the level of amylase and lipase which act as indicators of pancreas dysfunction.^115^The damage to pancreatic cells is associated with the development of acute diabetes.^115^ Studies support the expectation of endocrine system involvement in COVID-19 patients. SARS-CoV infection can cause dysfunction in the pancreas through immune-mediated mechanisms. The infection leads to the release of cytokines such as TNF-α–inducing apoptosis in pancreas cells.^115^ Also, SARS-CoV infection can result in impaired insulin sensitivity by increasing fetuin serum levels^115^ (Figure 2). Because of the high concentration of ACE2 in islets of the pancreas, any damages to islets by SARS-CoV-2 can lead to acute insulin-dependent diabetes.^115^ It is considered that insulin resistance can result from the adverse effects of lopinavir used for the treatment of COVID-19. According to the studies, lopinavir induces lipodystrophy which can be followed by subsequent insulin resistance.^115^

#### Obesity.

Obesity is an undeniable risk factor for COVID-19. According to studies, obese patients develop a severe form of the disease. Obesity can increase the intensity and severity of infections through different mechanisms.^116^ Regarding the expression of ACE2 receptors by the adipose tissue and the high concentration of the adipose tissue in obese patients, their susceptibility to be infected by SARS-CoV-2 is increased.^116^ Another mechanism is pertinent to a higher concentration of pro-inflammatory cytokines in COVID-19 patients. These pro-inflammatory cytokines are produced by the adipose tissue, and following an infection, they can cause dyslipidemia, insulin resistance, type 2 diabetes mellitus, hypertension, and cardiovascular disease.^116^ In addition, infection in obese patients can lead to cytokine storm which can induce severe diseases such as ARDS.^115^ Another mechanism is the high susceptibility of obese patients to develop thromboembolism which is a reason for the high mortality rate of obese COVID-19 patients.^116^

#### Thyroid gland dysfunction.

Thyrotoxicosis occurs because of direct affection of the thyroid gland by SARS-CoV-2, as described in other viral infections. Infection of the thyroid gland is known as subacute thyroiditis and is characterized by self-limiting thyrotoxicosis of variable duration lasting a period of time followed by hypothyroidism with the final restoration of euthyroidism.

#### Acute adrenal insufficiency and secondary adrenal insufficiency.

**Figure 4.**
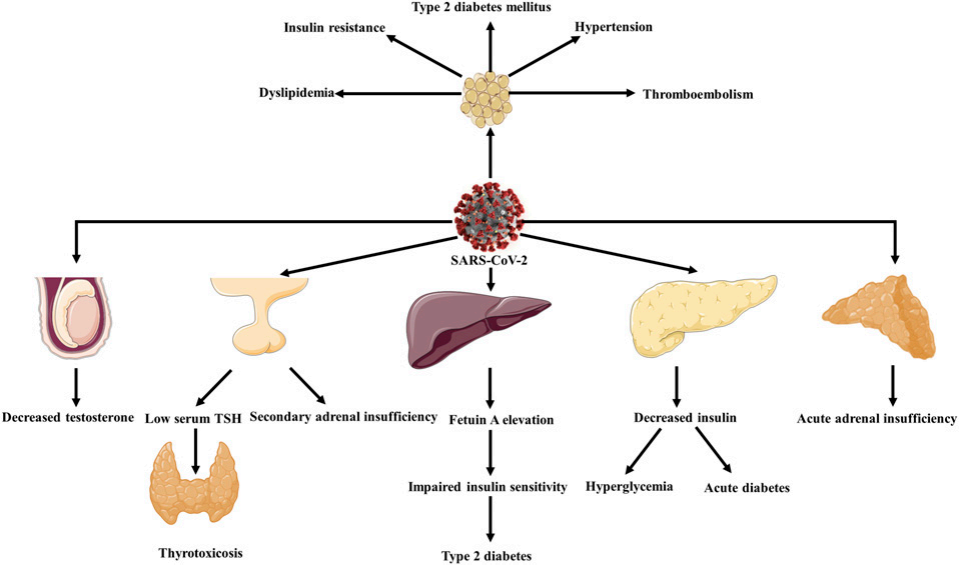
Endocrinological manifestations of SARS-CoV-2. Endocrine disorders such as acute diabetes, type 2 diabetes hyperglycemia, thyrotoxicosis and secondary adrenal insufficiency, and acute adrenal insufficiency are accounted for COVID-19 manifestations.

Possibility of VTE in COVID-19 patients is already reported. An acute adrenal insufficiency may be caused by thrombotic event at the adrenal level in COVID-19 patients. It can also cause an acute adrenal insufficiency with impaired hormone production with consequent shock and worsening of the possibility of reacting to severe respiratory distress^117^ (Figure 4).

According to autopsies, ACE2 receptors are expressed by hypothalamic and pituitary tissue; therefore, following SARS-CoV-2 infection, hypopituitarism is expected.^115^ Studies showed that in patients with SARS-CoV infection, secondary adrenal insufficiency occurs following infection.^118^ In these patients, SARS-CoV causes secondary adrenal insufficiency by inducing hypophysitis or by directly influencing the hypothalamus. Patients with secondary adrenal insufficiency present fatigue and dizziness. In patients with COVID-19, unexplained fatigue may be a result of the dysfunction of their hypothalamus–pituitary axis according to previous studies about SARS-CoV^118^ (Figure 4). However, further studies are required.

One of the primary immune invasive strategies used by SARS-CoV, as the influenza virus, is to knock down the host’s cortisol stress response.^119^ SARS-CoV expresses certain amino acid sequences that act as molecular mimics of the host adrenocorticotropic hormone (ACTH).^119^ SARS-CoV contains many permutations of amino acid sequences with homology to these probable ACTH key residues. Antibodies are produced by the host cells to counteract the virus and destroy the circulating ACTH as well.^115^ The demolition of ACTH decelerates cortisol rising. It can be inferred that numerous patients with SARS might have significant relative cortisol insufficiency. Because of the similar features of COVID-19 and SARS, cortisol insufficiency can be expected in COVID-19 patients.^119^ However, further studies are required. However, data on serum cortisol levels in patients with SARS and COVID-19 are not available until now. Besides, individuals with adrenal insufficiency have an increased rate of respiratory infection–related deaths, mainly due to improper immune function.^119^

## CONCLUSION

The rapidity of COVID-19 progression is correlated with extrapulmonary organ injuries and comorbidities. Considering the comorbidities and potential of organ injuries, the identification of various factors that account for multi-organ injuries and preventive and protective measures must be taken into serious consideration. Furthermore, these extrapulmonary manifestations are of particular importance to implement proper treatment strategies with personalized approaches to restrict the risk of decompensation. So, elucidation of poorly understood aspects of the disease would help to achieve success against the pandemic.
